# Molecular docking analysis of compounds from Justica adhatoda L with glycogen synthase kinase-3 β

**DOI:** 10.6026/97320630016893

**Published:** 2020-11-30

**Authors:** Selvaraj Jayaraman, Vishnupriya Veeraraghavan, Radhika Nalinakumari Sreekandan, Surapaneni Krishna Mohan, Sumetha Suga Deiva Suga, Devakumar Kamaraj, Sonaimuthu Mohandoss, Ponnulakshmi Rajagopal

**Affiliations:** 1 Department of Biochemistry, Saveetha Dental College and Hospitals, Saveetha Institute of Medical and Technical Sciences, Saveetha University, Chennai - 600 077, India; 2 Department of Clinical Skills & Simulation, Panimalar Medical College Hospital & Research Institute, Varadharajapuram, Poonamallee, Chennai - 600 123; 3 Department of Biochemistry and Department of Clinical Skills & Simulation, Panimalar Medical College Hospital & Research Institute, Varadharajapuram, Poonamallee, Chennai - 600 123; 4 Department of Microbiology, Panimalar Medical College Hospital & Research Institute, Varadharajapuram, Poonamallee, Chennai - 600 123; 5 Department of Pharmacology, Panimalar Medical College Hospital & Research Institute, Varadharajapuram, Poonamallee, Chennai - 600 123; 6 School of Chemical Engineering, Yeungnam University, Gyeongsan, Gyeongbuk-do, 38541, Republic of Korea; 7 Central Research Laboratory, Meenakshi Academy of Higher Education and Research (Deemed to be University), West K. K. Nagar, Chennai-600 078, India

**Keywords:** Type 2 Diabetes mellitus, Justica adhatoda L, molecular docking

## Abstract

Type 2 diabetes mellitus (T2DM) is linked with Glycogen synthase kinase-3 β.Therefore, it is ofinterest to document molecular docking analysis data of compounds from Justica adhatoda L with glycogen synthase kinase-3 β. We report the binding features of ethambutol, pyrazinamide, stigmasterol and vasicoline with GSK-3 β.

## Background

Type 2 diabetes mellitus (T2DM) accounts for 90% of the diabetic population and is correlated with obesity-induced insulin resistance accompanied by a high rate of insulin secretion from pancreatic β-cells. Glycogen synthase kinase-3 belongs to a super family of mitogen-activated protein kinases. GSK 3 was involved in the production of insulin resistance and the regulation of glycogen synthesis [1]. It is one of the main targets in the treatment of T2D. GSK-3 inhibitors provide antidiabetic properties as they enhance insulin sensitivity, glycogen synthesis and glucose metabolism in the skeletal muscles of diabetic patients [2]. Insulin receptors in peripheral tissues trigger a cascade of signaling pathways resulting in the activation of Akt, which subsequently allows phosphorylates to inhibit the function of GSK3 [3]. Insulin reduces the activity of GSK-3 [4]. GSK is also involved in ER stress in the β cells and decreases the function of GSK to protect the cells from death [5]. To date, several synthetic GSK-3 inhibitors have been developed. Very few experiments have been performed on plant-derived compounds with pharmacoinformatics for the detection of novel diabetic therapy [6]. However, due to their cytotoxicity, low pharmacokinetic and pharmacodynamic characteristics, clinical trials are restricted. Thus, the availability of accurate GSK-3 inhibitors especially from plant sources with a discreet mechanism of action on their substrates and structural studies provided crucial clues to the interactions shown by selective and non-selective ATP-competent GSK-3 inhibitors [7]. Justica adhatoda L. (Syn. Adhatoda Adhatodazeylanica Medicus) is grown in Indonesia, Malayasia, Southeast Asia, India and Pakistan [8] and has been an integral component in Unani medicine in India for some 200 years [9]. In addition, this plant has been used as an antispasmodic, anti-experimental, attenuating, fever-reducing, dying-fever, bronchial dilation, disinfectant, dysentery, bronchitis, whooping cough, glandular tumor, jaundice, diabetes, uterine constriction diarrhea, and certain liver-related disorders [10,11]. Over the years, a significant number of natural products have been identified from the genus Justicia which include: alkaloids, lignans, hormones, flavonoids, vitamins, irides, coumarins, diterpenoids, triterpenoids which triterpenoid glycosides [12-14]. Therefore, it is of interest to document molecular docking analysis data of compounds from Justica adhatoda L with glycogen synthase kinase-3 β.

## Materials and Methods:

### Ligand preparation:

The 12 compounds reported from Justicia adhatoda L have been retrieved from the pubchem database in SDF format (Table 1 - see PDF) [15] and converted into PDB file format using the Online Smile Translator. Energy minimization of ligands has indeed been carried out using ChemBio 3D Ultra 12.0, based on the process used.

### Protein preparation:

1Q4L X-ray crystal structure has been acquired from the Brookhaven Protein Data Bank (www.rcsb.org/pdb). Subsequent for screening process, the target protein (PDB Code: 1Q4L) has been chosen and prepared for molecular docking simulation in such a way that all heteroatomes (i.e. non-receptor atoms such as water, ions, etc.) have been eliminated.

### Molecular Docking:

Molecular docking analysis has been performed using the Autodock module available in PyRx Version 0.8 [16]. Blind docking has been executed to study insights into the molecular interaction between ligand and the target receptor protein. Blind docking was performed against the GSK-3 Beta (PDB ID: 1Q4L) protein data bank structure with compounds from Justicia adhatoda L. The size of the docking grid has been expanded to fit the entire protein within the grid with dimensions. Genetic Algorithm (GA) has been used for screening of the highest suitable blind docking conformers. Through molecular docking, maximum conformers have been considered for each compound to predict the best conformers in genetic algorithms. For each best one compound was selected for further interaction analysis.

### Molecular interactions visualization:

The auto dock vine generated docking pairs of proteins and ligands were retrieved in pdb format and examined in PyMOL [17] Visualization tool, a python-enhanced molecular graphics application. It also involves molecular editing, ray tracking, and recording. Binding sites and adjacent ligand amino acids have indeed been visualized. Molecular interactions in the form of hydrogen bonds between proteins and ligands have been reported and the distance of hydrogen bonds has also been calculated.

## Results and Discussion:

**Figure 1:**
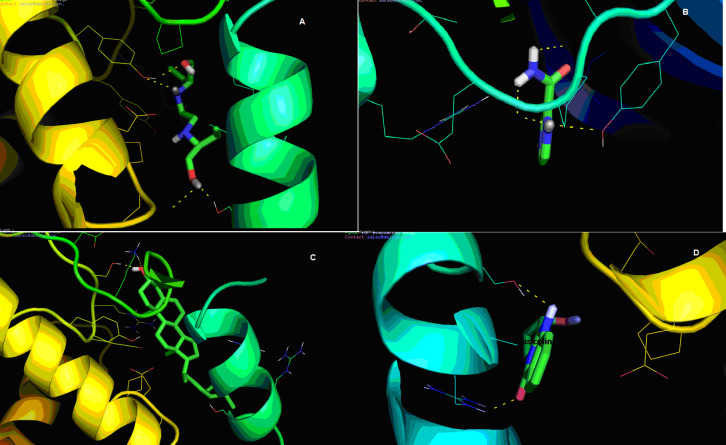
Interaction of GSK-3 with (a) Ethambutol; (b) Pyrazinamide; (c) Stigmasterol; (d) Vasicoline

Silicon molecular docking is a process that determines the desired orientation of a molecule to a second when it allows a stable compound to be bound together [18]. Even then, knowledge of the preferred orientation seems to be important to predict the strength of the relationship or affinity between the two molecules on the basis of their rating function. Generally, molecular docking is a complicated process consisting of two parts, such as the prediction and orientation of the ligand and the affinity to its target [19]. The relative orientation of the two interactive molecules, such as proteins, nucleic acids, carbohydrates and lipids, plays a key role in signal transduction, which has been found useful for predicting the intensity and form of signal produced. Thus, the minimum binding energy found during molecular docking plays a key role in drug discovery and development. The discovery and elucidation of GSK-3 in the control of different metabolic pathways results in the search for effective GSK inhibitors as candidates for the prevention and treatment of many chronic diseases [20]. So far, a number of molecules have also been synthesized to suppress the activity of glycogen synthase kinase. Even so, due to many disadvantages, none is considered to be optimal. The scope for novel GSK inhibitors, hopefully from plant origin with enhanced therapeutic and protection began to progress antidiabetic drugs. Docking simulations between the compounds of Justicia adhatoda L and glycogen synthase have been performed using the PyRx software; it's been shown to effectively produce good binding modes in terms of the minimum docking energy. The highest suitable binding modes of all ligands at the target protein active sites have been shown in (Figure 1a-d) using PYMOL v1.1. Hydrogen-binding ligands to four docked complexes and their corresponding energy values have been seen in (Table 2 - see PDF). GSK-3 could be blocked by three distinct mechanisms: I non-competitive ATP (substrate interaction domain), ii), competitive ATP (ATP binding pocket) and iii) competitive metal ion (Mg2 + binding site). GSK-3 was recognised as an essential kinase in the intercellular signaling pathway downstream of the insulin receptor. GSK-3 inactivates GS by phosphorylation resulting in inhibition of glycogen synthesis [7]. Some flavonoids, such as luteolin, rutin, narirutin, etc., have been documented to be able to bind to B-ring hydroxyls stabilized by hydrogen bonding with Arg 141 and Tyr 134 in the hinge. Our findings suggested that the selected four compounds could be formed by H-bonds or other interactions with the amino acid residues referred to above. As a result, these compounds displayed inhibiting GSK-3 activity. This research suggests that the Ethambutol with glycogen synthase kinase α complex is strengthened by three hydrogen bonds through the SER-147, TYR-222, and GLY-253 amino acid residues (Figure 1a) with a length of less than 3 Å. Interactions which play an important role in deciding the binding energy and stability of these receptor-ligand complexes have been known as hydrogen bonds. The binding energy of the least energy conformer of Ethambutol to glycogen synthase kinase 3ш complex has been calculated on a computational basis and had been found to be -6.8 Kcal / mol. (Figure 1b) shows the docking conformation of Pyrazinamide with glycogen synthase kinase 3β. The amino acids involved in the hydrogen bond interaction of glycogen synthase kinase 3β interacting with Pyrazinamide have been identified to be TYR-134, VAL-135, and PRO-136. The docking complex is strengthened at a distance of 2.3, 2.5, & 2.7Å, respectively. Pyrazinamide docking energy with glycogen synthase kinase 3β has been estimated to be - 5.6 Kcal / Mol.

The docking conformation of Stigmasterol with glycogen synthase kinase 35-007 was shown as (Figure 1c). The amino acids involved in the hydrogen bond interaction with glycogen synthase kinase 35-007 interacting with Stigmasterol were found to be LYS-183, & GLU-249, and the hydrogen bonding distance between Stigmasterol and 3α was found to be 21.6 & 1.5 Å. The docking energy for Stigmasterol with Glycogen synthase kinase 3α was calculated computationally and found to be - 5.5 Kcal/Mol. (Figure 1) d demonstrates the docking conformation of Vasicoline with glycogen synthase kinase 3β. Vasicoline with glycogen synthase kinase 3β complex is strengthened by bonded hydrogen bonds 2.0, and 2.5Å with residues ARG-144, and SER-147 of GSK 3β, respectively. The binding energy for Vasicoline to Glycogen synthase kinase 3β was found to be-7.1 Kcal / Mol. The parameters studied in this study include hydrogen bond interactions, binding energy, and orientation of phytochemicals to receptors. These would be considered to be significant molecular docking indices. If a compound has less binding energy, it means that perhaps the compounds have greater efficacy. The magnitude of the attraction or repulsion forces between the ligand and the receptor depends on the binding or intermolecular energy. Accordingly, the findings of this study have indicated that perhaps the decrease in binding energy as well as the inhibition constant of the four bioactive compounds (ligands) seems to be responsible for their GSK inhibitory action.

## Conclusion

We report the binding features of ethambutol, pyrazinamide, stigmasterol and vasicoline with GSK-3 β for further consideration in the context of T2DM.
